# Successful treatment of refractory eosinophilic annular erythema with tofacitinib^؆^

**DOI:** 10.1016/j.abd.2025.501194

**Published:** 2025-08-18

**Authors:** Ying-Luo Niu, Hai-Yang He, Sheng Fang

**Affiliations:** Department of Dermatology, the First Affiliated Hospital of Chongqing Medical University, Chongqing, China

Dear Editor,

Eosinophilic Annular Erythema (EAE) is a rare eosinophilic dermatosis characterized by annular erythematous plaques with varying degrees of pruritus. Treatment options for EAE are often limited and the disease is often refractory with a recurrent course. Highlighting the potential of tofacitinib in the treatment of EAE, we report a case of refractory EAE that was effectively treated with tofacitinib.

A 34-year-old female was admitted to our department with pruritic annular plaques on the trunk and proximal extremities. The patient had no history of food or drug allergies or other chronic diseases. Physical examination revealed scattered dark red annular plaques with raised borders on the neck, anterior chest, waist, and abdomen (Fig. 1A‒D). Oral hydroxychloroquine and antihistamines were given for two weeks with no response. Laboratory tests revealed an elevated eosinophil count (0.78 × 10^9^/L), while no abnormalities were observed in liver enzymes, renal function, urinalysis, total serum IgE levels, antinuclear, anti-DNA, and HIV tests. A skin biopsy showed a superficial perivascular infiltration composed predominantly of eosinophils. There was no evidence of vasculitis, flames figures, dermal mucin, or vacuolar changes. Direct immunofluorescence was negative. Given the clinical presentation and the lack of flame figures, Wells syndrome was excluded. The diagnosis of EAE was made on the basis of the above findings. Monotherapy with tofacitinib 5 mg twice daily was started. After one week of treatment, the patient showed remarkable improvement. The annular erythema became lighter in color, blurred in outline and the raised margins disappeared (Fig. 1B‒E). She was treated for a further two weeks. At a follow-up visit four months after treatment, we observed that the lesions were almost completely cleared without recurrence (Fig. 1C‒F). The histopathological characteristics are shown in Fig. 2 (Fig. 2A‒B). In a follow-up phone call after 8 months, the patient was completely cured and there was no relapse.

**Fig. 1:**
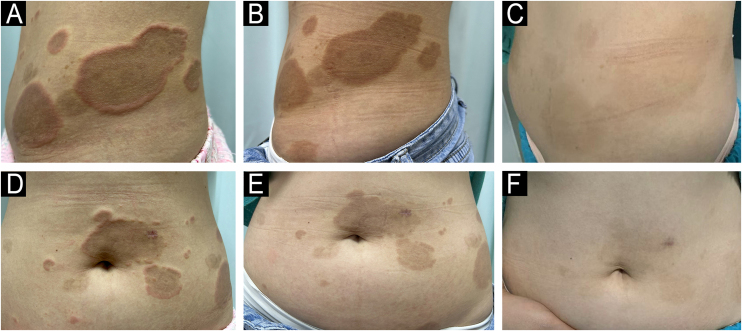
Clinical image of the patient treated with tofacitinib. (A‒D) The patient presented with scattered dark red annular plaques with raised borders on the truck before treatment. (B‒E) Remission of clinical manifestations after treatment with tofacitinib in a week. (C‒F) The lesions were completely cleared without any recurrence in a 4-month-follow up.

**Fig. 2:**
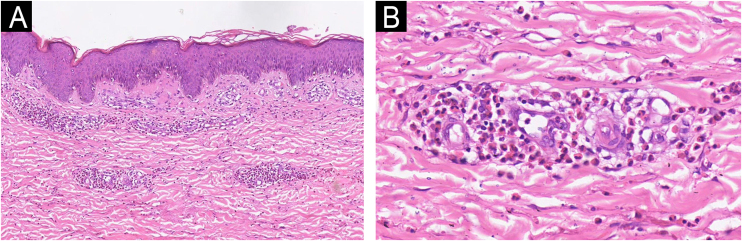
(A) Histopathology showed a superficial perivascular infiltration (Hematoxylin & eosin, ×100). (B) A high-power field showed perivascular infiltration composed predominantly of eosinophils (Hematoxylin & eosin, ×400).

EAE is a rare self-limited eosinophilic dermatosis with a relapsing and remitting course. It can affect individuals of all ages, though it is more commonly seen in young adults. While the exact incidence remains unclear, EAE has been associated with drug reactions, allergic responses, and underlying systemic conditions, such as systemic lupus erythematosus and eosinophilic gastroenteritis. An interesting case report highlights a potential association between EAE and Primary Biliary Cholangitis (PBC), a systemic disease that has not been previously reported in the literature.^1^ The differential diagnosis of EAE includes Wells syndrome, rheumatoid erythema annulare, erythema annulare centrifugum, and other eosinophilia-associated skin diseases, and some authors consider it to be a subtype of Wells syndrome.^2^ Wells' syndrome and EAE are two eosinophil-associated dermatoses that share some similarities in clinical presentation and histopathology, but there are important differences. Wells' syndrome presents as erythematous, edematous plaques that resemble cellulitis, and the lesions may be accompanied by pruritus or pain. The dermis is heavily infiltrated with eosinophils, with characteristic “flame figure”, while lesions of EAE are circular or arcuate erythematous plaques with raised margins and fading in the center, and the lesions are usually asymptomatic or mildly pruritic, with eosinophilic infiltration of the dermis, but without the “flame figure”.

Due to the rarity of EAE, there is no standard treatment for the disease. Currently, first-line therapies are glucocorticoids, antihistamines, chloroquine, and hydroxychloroquine. Alternative treatment options include ciclosporin, mepolizumab, dapsone, narrowband UVB, doxycycline, and benralizumab (Table 1).^3^ However, the treatment of EAE remains a challenge due to the high relapse rate and unsatisfactory therapeutic efficacy.

**Table 1:** Alternative treatment options for EAE and the outcome.

Treatment	Treatment Mechanisms	Reported Outcomes	Administration	Potential Side Effects
Ciclosporin	Calcineurin inhibitor, suppresses T-cell activation	Effective in reducing inflammation and improving symptoms in some cases	Oral	Hypertension, nephrotoxicity
Upadacitinib	JAK1 inhibitor, reduces immune cell activation	Reduced disease activity and inflammation; limited data in EAE	Oral	Headache, upper respiratory infections
Mepolizumab	IL-5 inhibitor, reduces eosinophil counts	Decreased eosinophil infiltration and inflammation; limited evidence for EAE	Subcutaneous, monthly	Headache, nasopharyngitis
Dapsone	Anti-inflammatory, antimicrobial properties	Improvement in skin symptoms in EAE; limited data on systemic improvement	Oral	Hemolysis, methemoglobinemia
Narrowband UVB	UV radiation, reduces skin inflammation	Effective for skin lesions in EAE; minimal evidence for systemic benefit	Topical	Skin irritation, erythema
Doxycycline	Antibiotic with anti-inflammatory effects	Possible benefits for chronic inflammation; limited evidence in EAE	Oral	Gastrointestinal upset, photosensitivity
Benralizumab	IL-5 receptor antagonist, reduces eosinophil counts	Significant eosinophil reduction; potential benefit in EAE-related inflammation	Subcutaneous, every 4 weeks	Headache, upper respiratory tract infections

EAE has not been clearly elucidated, but hypersensitivity reactions to unidentified antigens have been proposed, while aberrant tissue-resident eosinophils may participate in the disease. Tofacitinib selectively inhibits JAK1 and JAK3 to modulate immune cell activity and reduce inflammation. It inhibits the activation of T cells and B cells through the JAK-STAT pathway. It can suppress the production of several inflammation-related cytokines such as IL-4, IL-5 and IL-13, which play critical roles in proliferation, activation, and recruitment of eosinophils.^4^, ^5^, ^6^ Recent studies suggested that inhibiting JAK-STAT signaling, especially JAK1 signaling, can both suppress Th2 differentiation and eosinophil activation.^7^ As the JAK-STAT pathway mediates important cellular processes such as immune response and inflammation, tofacitinib can be used as a targeted therapy by blocking these pathogenic pathways.^6^

To conclude, to the best of our knowledge, we first reported a case of refractory EAE successfully treated with tofacitinib. Our observation supported that JAK inhibitors may be a promising therapy option and shed a bright light on the treatment of EAE. However, further studies are needed to provide more evidence and confirm the findings.

## Research data availability

The data that support the findings of this study are available from the corresponding author upon reasonable request.

## Scientific associate editor

Hiram Larangeira de Almeida Jr.

## Financial support

None declared.

## Authors' contributions

Ying-Luo Niu: Collected clinical data, including images and medical records, and drafted the manuscript; participated in the diagnostic discussion and contributed to case interpretation.

Hai-Yang He: Assisted with data collection; participated in the diagnostic discussion and contributed to case interpretation.

Sheng Fang: Supervised the project and provided critical revisions; participated in the diagnostic discussion and contributed to case interpretation.

## Conflicts of interest

None declared.
